# Experience of general anesthesia in a patient with menstrual‐associated coronary spasm

**DOI:** 10.1002/ccr3.7641

**Published:** 2023-07-05

**Authors:** Aya Kawachi, Saho Sudo, Yusuke Ishida, Koichi Nakazawa

**Affiliations:** ^1^ Department of Anesthesiology Tokyo Medical University Tokyo Japan

**Keywords:** coronary spasm, general anesthesia, menstruation

## Abstract

Coronary angina due to low estrogen is relatively common around menopause, with almost no reports associated with the menstrual cycle or anesthetic management at younger ages. The patient was a 22‐year‐old woman who had developed ventricular fibrillation due to coronary spasm, resulting in cardiopulmonary arrest. She was resuscitated, and underwent ICD implantation. As her symptoms appeared at specific times during her menstrual cycle, she was diagnosed as having menstrual‐associated coronary spasm, and started taking estrogen/progesterone medication. An endometrial ablation was scheduled for endometrial hyperplasia that was caused by the medicine. The surgery was scheduled in consideration of the patient's menstrual cycle, and general anesthesia was selected as the method of anesthesia. The surgery and perioperative management were uneventful, and her postoperative course was favorable. Our case is the first to our knowledge of general anesthesia performed on a patient with menstrual‐associated coronary spasm.

## 1 BACKGROUND

Coronary spasm is a rare condition that occurs in premenopausal women in association with phases of the menstrual cycle, and is thought to be associated with the decrease in estrogen levels. To our knowledge, there have been no reports to date of anesthesia management of patients with menstrual‐associated coronary spasm. We encountered a case of a patient with a history of coronary spasm induced by hormonal changes in the menstrual cycle, who was treated with intraoperative prophylaxis, and appropriate anesthesia to prevent coronary spasm attacks during surgery for endometrial ablation. We here report a review of the anesthetic management of this rare case.

## 2 CASE PRESENTATION

The patient was a 22‐year‐old woman (height: 165 cm; weight: 86.5 kg; BMI: 31.8 kg/m^2^). She was scheduled to undergo endometrial ablation for endometrial hyperplasia. Regarding her medical history, she experienced a cardiopulmonary arrest owing to ventricular fibrillation at the age of 15, at which time she was diagnosed as having coronary spasm via a coronary angiogram and acetylcholine stress test. She underwent ICD implantation, together with the initiation of treatment with a Ca^2+^ blocker and coronary dilators. The ICD was activated four times in 6 years, and the patient resumed a self‐paced heartbeat. After a careful interview regarding the circumstances of the onset of her symptoms, we strongly suspected menstrual‐associated coronary spasm, as her symptoms occurred from just before menstruation to the middle of menstruation. Estrogen/progesterone replacement therapy was started, and her ICD activation and angina attacks ceased. When she was 22 years old, we decided to perform an endometrial ablation with tissue biopsy for endometrial hyperplasia that was thought to be caused by the estrogen/progesterone medication. Her family history included coronary angina pectoris in the father. Her oral medications were diltiazem (120 mg/day), nifedipine (40 mg/day), nicorandil (25 mg/day), and isosorbide mononitrate (40 mg/day), and an isosorbide mononitrate patch (80 mg/day). She had no history of smoking. Her first menstrual period was at 11 years old, and her menstrual cycle was 30 days and regular. Her last menstrual period was 14 days before the scheduled surgery. Blood test findings were normal. Chest X‐ray displayed no cardiac enlargement or abnormal shadows, except for the presence of an ICD device. Preoperative estradiol levels were 63.1 pg/mL in the follicular phase, and 42.9 pg/mL in the ovulatory phase, which were within the normal ranges for these phases in nonpregnant women. Her ECG results were normal, with a pulse of 55 beats/min, sinus rhythm, and a corrected QTc of 403 ms. Transthoracic echocardiography displayed an ejection fraction of 65%, no abnormal wall motion, no systolic or diastolic dysfunction, and no apparent valvular disease. There were load‐induced right ventricular abnormalities, and she had a NYHA classification of grade I. Anesthesia was induced with remifentanil (0.3 μg/kg/min) and propofol TCI (4.0 μg/mL), and muscle relaxation was achieved with rocuronium bromide (50 mg). After endotracheal intubation using a McGrath MAC® video laryngoscope (Medtronic Co., Minneapolis, MN, USA), anesthesia was maintained with propofol TCI (4.0 μg/mL), continuous infusion of remifentanil (0.1–0.2 μg/kg/min), and rocuronium bromide 50 mg, and sedation levels were monitored using the patient state index measured with a Sedline® monitor (Masimo Co.), and maintained at scores between 30 and 50. In addition, an observation arterial pressure line was taken. Intraoperative and postoperative nicorandil (1 μg/kg/min) and diltiazem (1 μg/kg/min) were continuously administered intravenously. During surgery, the ICD was deactivated and a percutaneous external defibrillator was used.

The patient remained unchanged and her blood pressure did not decrease, and no antihypertensive or antiarrhythmic medications were administered during the surgery. Surgery time was 13 min, and anesthesia time was 49 min. Bleeding was minimal. The surgery was completed without any complications, and the patient was extubated and returned to the ICU after surgery. The patient was discharged on the day after the surgery.

## 3 DISCUSSION

Coronary spasm is defined as a transient abnormal contraction of the coronary arteries. Factors known to contribute to the onset of the disease include smoking, alcohol consumption, abnormal lipid and sugar metabolism, stress and hyperventilation, genetic factors, and female hormone deficiency.^1^ The female hormone estrogen is known to have cardioprotective effects.^2^ The mechanism is thought to be vasodilation via estrogen receptors in vascular endothelial cells, vascular smooth muscle cells, and the heart, suppression of neointimal proliferation, and suppression of angiotensin 1 receptor expression, which has a myocardial hypertrophic effect.^3^, ^4^, ^5^ Premenopausal women have a late luteal to menstrual phase when blood estrogen levels are low, and a follicular and ovulatory phase, and early luteal phase when estrogen levels are high. For this reason, it has been noted that coronary spasms and acute coronary syndromes are more common during the late luteal phase and menstrual phase, when estrogen levels are low.^6^ In the present patient, life‐threatening arrhythmias due to coronary spasm occurred during the luteal phase to the menstrual phase.

The frequency of coronary spasm attacks and anginal symptoms improved after treatment with estrogen (Duphaston®) and progesterone (Premarin®) was started. Sugiura et al. reported that oral contraceptives and low‐dose estrogen/progesterone products, regardless of their type, are associated with a significant increase in deep vein thrombosis.^7^ Perioperative anticoagulation was not performed in this case because the surgery was small and the patient was ambulatory the day after surgery. In the perioperative period, it is recommended that medication be withdrawn 4 weeks prior to surgery for surgeries lasting longer than 45 min, and postoperatively until immobility is resolved.^8^ In the present case, both drugs were withdrawn 30 days prior to surgery. Considering that the scheduled surgery time for this case was 30 min, and the patient was scheduled to be discharged the day after surgery, there may have been no need for drug withdrawal. The timing of the surgery was appropriate because it was scheduled around the time of ovulation, 2 weeks after the last menstrual period. Problems with estrogen/progesterone therapy include an increased risk of breast cancer owing to high progesterone levels,^9^ increased incidence of coronary vascular disease, and abnormal vaginal bleeding. However, it has been pointed out that estrogen monotherapy increases the risk of developing endometrial hyperplasia and endometrial cancer. The frequency of endometrial hyperplasia coexisting with or progressing to cancer is reported to be about 1%–30%,^10^ and if the patient is required to take oral contraceptive pills from a young age, as in this patient, the risk of developing cancer in the future may be high. In the present patient, intravenous diltiazem hydrochloride and nicorandil were started at the time of induction of anesthesia for the prevention of coronary spasms, and was continued during and after surgery, until just before discharge. According to the Guidelines for the Diagnosis and Treatment of Coronary Angina Pectoris, the Ca^2+^ antagonist diltiazem hydrochloride is extremely effective in preventing coronary spasms, and is recommended as class I drug. Nicorandil has selective coronary vasodilation and anticoronary spasm effects, and is classified as a class II drug that is recommended with a high likelihood of efficacy when used in combination with a Ca^2+^ antagonist for seizures that are difficult to control with the antagonist alone.^11^

There have been no reports on the anesthesia of patients with menstrual‐associated coronary angina pectoris. The present patient required an endometrial ablation, and the attending physician, cardiologist, and anesthesiologist discussed whether the procedure should be performed under general anesthesia or spinal anesthesia.

Anesthesia management with spinal anesthesia was initially considered, but there were concerns regarding side effects from the use of various drugs, and the risk of suppression of cardiac function. However, there have been many previous reports of coronary spasms induced by relative parasympathetic dominance due to sympathetic blockade by epidural or spinal anesthesia^12^ ^.^ ^13^Therefore, in this case, the patient was managed under general anesthesia, considering the possibility that coronary spasms might occur due to sympathetic blockade by spinal anesthesia.

## AUTHOR CONTRIBUTIONS

**AYA KAWACHI:** Writing – original draft; writing – review and editing. **Saho Sudo:** Writing – review and editing. **Yusuke Ishida:** Writing – review and editing. **Koichi Nakazawa:** Writing – review and editing.

## FUNDING INFORMATION

None.

## CONFLICT OF INTEREST STATEMENT

The authors declare that they have no competing interests associated with this manuscript.

## ETHICS STATEMENT

Not applicable.

## CONSENT STATEMENT

Written informed consent was obtained from the patient for publication of this case report and the accompanying images.

## Data Availability

Not applicable.
